# The prevalence of human papillomavirus and its impact on cervical dysplasia in Northern Canada

**DOI:** 10.1186/1750-9378-8-25

**Published:** 2013-07-01

**Authors:** Ying Jiang, Paul Brassard, Alberto Severini, Yang Mao, Y Anita Li, Julie Laroche, Susan Chatwood, Andre Corriveau, Kami Kandola, Brendan Hanley, Isaac Sobol, Muna Ar-Rushdi, Gordon Johnson, Jane Lo, Sam Ratnam, Tom Wong, Alain Demers, Gayatri Jayaraman, Stephanie Totten, Howard Morrison

**Affiliations:** 1 Science Integration Division, Public Health Agency of Canada (PHAC), 785 Carling Ave., Ottawa, ON, K1A 0K9, Canada; 2 Division of Clinical Epidemiology, McGill University Health Center, 687 Des Pins West, Montreal, QC, H3A 1A1, Canada; 3 National Microbiology laboratory, PHAC, 1015 Arlington St., Winnipeg, MB, R3E 3R2, Canada; 4 Centre for Immunization and Respiratory Infectious Diseases, PHAC, 130 Colonnade Rd, Ottawa, ON, K1A 0K9; 5 Institute for Circumpolar Health Research, 3506 MacDonald Road, Yellowknife, NWT, X1A 3X7, Canada; 6 Department of Health & Social Services, CST-6, Yellowknife, NWT, X1A 2L9, Canada; 7 Department of Health & Social Services, #4 Hospital Road, Whitehorse, YK, Y1A 3H8, Canada; 8 Nunavut Department of Health and Social Services, Nunavut (at the time of the data collection) and currently with Health Canada, Nunavut, Canada; 9 Labrador-Grenfell Health, Happy Valley-Goose Bay, NL, Canada; 10 Dynacare Kasper Medical Laboratories, 200.10150 102 Street, Edmonton, AB, T5J 5E2, Canada; 11 Provincial Health Services Authority Laboratories, British Columbia Cancer Agency, 686 West Broadway, Vancouver, BC, V5Z 1G1, Canada; 12 Faculty of Medicine, Memorial University, St. John’s, NL, Canada; 13 Centre for Communicable Diseases and Infection Control, PHAC, 200 Eglantine Driveway, Tunney’s Pasture, Ottawa, ON, K1A 0K9, Canada

**Keywords:** Human papillomavirus, Prevalence, Pap abnormality, Northern region

## Abstract

**Introduction:**

Certain types of the Human Papillomavirus (HPV) are sexually transmitted and highly associated with development of cervical dysplasia and cervical cancer but the distribution of HPV infection in the North, particularly amongst First Nations, Metis, and Inuit peoples, is little known. The purposes of the study are to identify the prevalence of type-specific HPV infections and the association of different HPV types with cervical dysplasia among women in Northern Canada.

**Methods:**

This was a cross-sectional study with attendants of the routine or scheduled Pap testing program in the Northwest Territories (NWT), Nunavut, Labrador and Yukon, Canada. Approximately half of each sample was used for Pap test and the remaining was used for HPV genotyping using a Luminex-based method. Pap test results, HPV types, and demographic information were linked for analyses.

**Results:**

Results from 14,598 specimens showed that HPV infection was approximately 50% higher among the Aboriginal than the non-Aboriginal population (27.6% vs. 18.5%). Although the most common HPV type detected was HPV 16 across region, the prevalence of other high risk HPV types was different. The age-specific HPV prevalence among Aboriginal showed a ‘U’ shape which contrasted to non-Aboriginal. The association of HPV infection with cervical dysplasia was similar in both Aboriginal and non-Aboriginal populations.

**Conclusions:**

The HPV prevalence was higher in Northern Canada than in other Areas in Canada. The prevalence showed a higher rate of other high risk HPV infections but no difference of HPV 16/18 infections among Aboriginal in comparison with non-Aboriginal women. This study provides baseline information on HPV prevalence that may assist in surveillance and evaluation systems to track and assess HPV vaccine programs.

## Introduction

Cervical cancer is the second most common cancer of the women worldwide. In Canada invasive cervical cancer and associated mortality among Aboriginal women have been 2–4 times higher than among non-Aboriginal women [1-4]. From the Statistics Canada 2006 census Aboriginal groups make up more than 50% of the population in the Northwest Territories (NWT) [5] (First Nations, Metis, and Inuit), 85% of the population in Nunavut [6] (predominantly Inuit), 35% in Labrador [7] (Metis, Inuit, and First Nations) , and 25% in the Yukon [8] (predominantly First Nations). Increased risk of cervical cancer in Aboriginal women has been consistently observed in various regions across Northern Canada [2,9,10]. The gap in cervical cancer and associated mortality between the Aboriginal and non-Aboriginal groups has important implications for cancer control policy. Implementation and evaluation of cervical cancer prevention programs in Aboriginal women may require a different approach.

Human Papillomavirus (HPV) is a common sexually transmitted infection worldwide. Persistent infection with specific types of the virus is necessary for the development of invasive cervical cancer and its precursor lesions [11]. HPV is detected in almost 100% of women with invasive cervical cancer and types 16 and 18 are responsible for approximately 70% of all cases [12,13]. In Canada HPV 16/18 prevalence has been reported in 56.2% of high grade precursor lesions [14].HPV 16/18 prevalence can vary by geography and ethnicity. Therefore, determination of type-specific HPV prevalence in a region may be one of the important steps towards cervical cancer prevention.

Only very limited regional data [2,3,15] provide HPV prevalence in Northern Canada which appears different with that of the general population. Population based research on HPV and related outcomes in the North could fill this information gap. For cervical cancer prevention and early detection of pre-cancerous lesions, routine Papanicolaou smear (Pap) test screening is offered in all regions of Northern Canada with good coverage of targeted population. In 2008, 88%, 74%, 83%, and 81% of women aged 18–69 in NWT, Nunavut, Labrador, and Yukon respectively, were reported as having had a Pap test in the previous three years [16]. It was therefore considered a practical and efficient approach to examine HPV prevalence as part of routine Pap smear screening.

The purpose of this study is to measure the prevalence of HPV infections by type and region and to determine their impacts on cervical dysplasia across Northern Canada.

## Methods

### Study population

This was a cross-sectional study including all women aged 14 years or older who presented for a regular Pap test in the four regions of Northern Canada: NWT (April, 2008 - March, 2009), Nunavut (January, 2008 - March, 2009), Labrador (February - November, 2010), and Yukon (March, 2009 - June, 2010). Women with a cervical cancer history and those who chose to opt-out were excluded from the study. Only the first sample collected was kept for analysis for women with more than one Pap smear performed over the study period. An opt-in consent process was used in Yukon which was different with the other jurisdictions. Given the universal coverage of the health care system in Canada there are no other Pap test providers in these areas.

### Authority and licensing

This research received ethical approval from the Health Canada Ethics Review Board, McGill University Ethics Review, and the local health authorities including NWT Department of Health and Social Services, Stanton Territorial Health Authority, the Yukon Department of Health and Social Services, and the Yukon Scientists and Explorers Act License. A Northern research licence was also obtained from Aurora Research Institute in the NWT. The project was facilitated by four data collection sites and the manuscript was reviewed by community stakeholders before being submitted for publication.

### Data source

Pap test screening is performed in each Territory and the Labrador health region in Canada. It is offered to all women aged 18–69 and young women aged 14–18 who wish to participate. Pap smears are collected at community health centres, medical clinics, or hospitals whichever appropriate. Pap test registration form is used to assemble personal record and demographic information on site. Demographic details including Aboriginal status, age, and region information were abstracted from the routine Pap test registration form. Aboriginal status was only available among NWT, Nunavut, and Yukon datasets. Only area code (residence in Aboriginal area or non-Aboriginal area) was available in the Labrador dataset. Data were collected, organised, and owned by the local health authorities.

### Specimen collection and preparation

Samples from NWT and Nunavut were collected in liquid based cytology (LBC) medium (SurePath®) and were shipped to the DynaLife Medical Laboratory in Edmonton, where cytology was read and remaining half of the specimen was kept for HPV testing. Samples from Labrador were also collected in SurePath LBC medium and shipped to Newfoundland Public Health Laboratory for Pap test interpretation. In Yukon, conventional Pap smear slides were taken and shipped to the Cervical Cancer Screening Laboratory, BC Cancer Control Agency. A sample for HPV testing was prepared by taking a second cervical sample and by rinsing the brushes in LBC medium. Samples for HPV genotyping were shipped from all four areas to the National Microbiology Laboratory (NML) in Winnipeg, Canada. Cervical cytology and HPV testing were done independently to eliminate the potential for reporter bias.

### HPV typing

For HPV genotyping an “in house” Luminex assay [17] developed at the NML was used. This assay detects 45 high risk and low risk HPV types. Comparison against the Linear Array (Roche) [17] and other HPV genotyping kits [18] showed that this Luminex assay is comparable to other commercial genotyping methods. The 45 HPV types included 23 of the 25 IARC high risk (HR) types found in group 1, 2a, and 2b (HPV 16, 18, 26, 30, 31, 33, 35, 39, 45, 51, 52, 53, 56, 58, 59, 66, 67, 68, 69, 70, 73, 82 and 85) [19], and other 22 types considered of low (LR) or unknown risk (6, 11, 13, 32, 40, 42, 43, 44, 54, 61, 62, 71, 72, 74, 81, 83, 84, 86, 87, 89, 90, and 91) [19].

### Pap smear testing

Cytological reports were issued according to the Bethesda system [20,21]. The results were classified as: normal (including benign), atypical squamous cells of undetermined significance (ASC-US), low-grade squamous intraepithelial lesion (LSIL), and high-grade squamous intraepithelial lesion (HSIL). Samples with atypical squamous cells (ASC-H), where HSIL could not be excluded, were added to the HSIL category. To be consistent with reported data worldwide we classified LSIL and HSIL as abnormal categories for the correlation analysis and impact assessment. ASC-US cases were not included in this part of analysis.

## Statistical analyses

### Data preparation

Demographic information, HPV types and Pap test results were linked for HPV prevalence analysis. In the linkage dataset, 764 records (approximately 5% of collected data in the study) with one or more items of missing value were excluded from the analysis; 485 missing in age or age-group, 327 in ethnic status or ethnic area, and 399 in Pap test including 126 unsatisfactory cytology results.

HPV_s and HR_s were defined as cases with only a single type of HPV infection and cases with only a single type of high risk HPV infection, respectively. Similarly, HPV_m and HR_m were defined as samples with two or more types of HPV or HR HPV infection. Samples with two or more HPV types were counted in for each type in type-specific analysis.

### Analysis

Prevalence rates for type-specific HPV infection and cervical dysplasia were computed. Based on the 2008 census data among women in the NWT region [5] as the standard, the age-adjusted prevalence rates (AAPR) were calculated for area comparison. The overall prevalence rate in Northern Canada was also weighted by population size [5-8] by region.

A multivariate logistic regression analysis was used to explore the associations between HPV infection and cervical dysplasia, adjusted for Aboriginal status, age, and region. Odd Ratios (ORs) were calculated by different level: (a) infection with any HPV type, single HR infection, multiple HR infection, single LR infection, multiple LR infection, and infection with both LR and HR types, relative to no HPV infection, and (b) infection with both HR and LR HPV types relative to a single LR HPV infection.

The proportion of cervical dysplasia caused by HPV infection in Northern Canada was measured by the exposure attributable risk fraction (ARF) and the population attributable risk fraction (PAR). In calculating the PAR, to avoid duplicate counting of those infected with both HR and LR, the exposed population was first stratified into those infected with only HR, only LR, and both. The PARs were summed across the strata. ORs are used in the calculation of both ARF and PAR: ARF = (OR-1)/OR and PAR = (Number of exposed cases x ARF)/total cases in the population. Furthermore, PAR of HR HPV infection = (No. of HR_s cases x ARF reference to HPV negative + No. of only multiple HR cases x ARF reference to HPV negative + No. of both HR and LR infected cases x ARF reference to LR HPV) / total cases [22].

All data management and analyses were carried out using Statistical Analysis System 9.1. Two-tailed p value <0.05 was the criteria to determine the statistical significant [23].

## Results

A total of 14598 cytology samples were collected and tested from NWT (n=6940), Nunavut (n=4683), Labrador (n=1370), and the Yukon (n=1605). In NWT and Nunavut the study covered more than 95% of the Program targeted population, respectively; but only 77% and 27% in Labrador and the Yukon in the study period. In this study, approximately 42% of women were non-Aboriginal and 58% were Aboriginal. The overall mean and median ages of participants were 35 and 32, respectively.

### HPV prevalence analysis

#### Type specific

**Figure 1:**
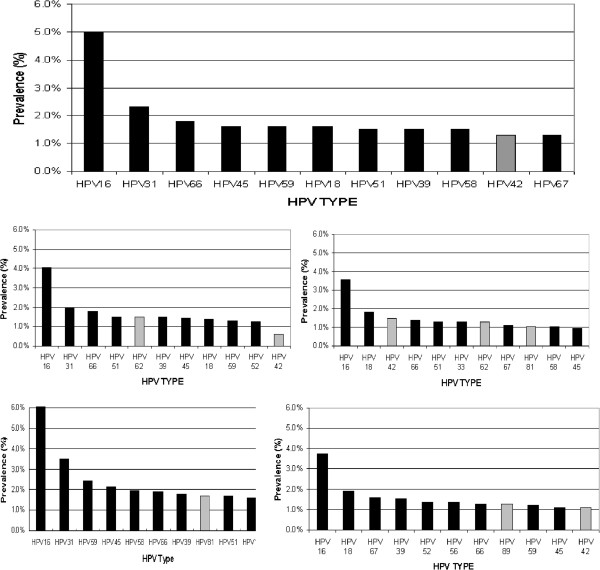
HPV infection type distribution in NWT, Nunavut, Labrador and Yukon, Canada.

HPV-DNA was detected in 26% of subjects (Table 1). After weighting the four regional population sizes from census data, the overall prevalence rate in Northern Canada was estimated to be 25.2%. Although the specific prevalence of HPV types was dissimilar among regions, the highest prevalence HPV types detected were HPV-16 (5.0%), HPV-31 (2.3%), HPV-66 (1.8%), HPV-45 (1.6%), HPV-59 (1.6%), and HPV-18 (1.6%). HPV type distributions are different by region (Figure 1). Among HPV infected samples, 79% had an HR type, 33% had multi-types, and 25% had HPV 16/18. The overall prevalence of HPV_m, HR_s, HR_m, and LR_s were 8.4%, 13%, 4.0%, and 4.7%, respectively.

**Table 1:** **HPV type distribution by cytological results in Northern Canada**^1^

**HPV**^2^**typing**	**Cytological results**^3^				**Total**	
	**Normal**	**ASC**-**US**	**LSIL**	**HSIL**+**ASC**-**H**	**n**	**%**
**HPV negative**	10488	92	46	19	10645	74.1%
**HPV positive**	2891	320	372	145	3728	25.9%
**Total**	13379	412	418	164	14373	100.0%
**HR**-**HPV**						
16^3^	485	79	85	71	720	5.0%
31	236	44	31	17	328	2.3%
66	172	30	50	2	254	1.8%
45	171	27	23	10	231	1.6%
59	179	20	26	6	231	1.6%
18	141	32	41	10	224	1.6%
51	151	17	47	4	219	1.5%
39	153	18	40	8	219	1.5%
58	148	21	27	15	211	1.5%
67	142	16	18	6	182	1.3%
52	132	21	13	12	178	1.2%
70	131	12	21	2	166	1.2%
56	99	8	36	5	148	1.0%
33	94	15	24	9	142	1.0%
35	85	9	16	11	121	0.8%
73	59	6	10	1	76	0.5%
53	48	7	18	1	74	0.5%
68	35	3	4	1	43	0.3%
30	20	7	5	0	32	0.2%
69	24	2	4	0	30	0.2%
85	24	3	3	0	30	0.2%
82	11	2	5	0	18	0.1%
26	3	2	3	1	9	0.1%
Total	2743	401	550	192	3886	27.0%
**LR**-**HPV**						
42	159	10	14	2	185	1.3%
62	158	9	7	0	174	1.2%
81	129	12	11	2	154	1.1%
6	112	16	22	1	151	1.1%
89	107	9	9	0	125	0.9%
72	101	2	3	1	107	0.7%
54	94	3	9	0	106	0.7%
83	75	4	6	0	85	0.6%
84	70	7	3	0	80	0.6%
40	55	6	6	2	69	0.5%
74	62	6	1	0	69	0.5%
90	53	2	12	0	67	0.5%
86	43	3	1	0	47	0.3%
11	27	9	8	0	44	0.3%
44	48	1	0	0	49	0.3%
43	15	2	5	1	23	0.2%
32	19	1	0	1	21	0.1%
87	18	0	1	0	19	0.1%
13	10	3	2	0	15	0.1%
61	9	1	0	0	10	0.1%
91	2	1	5	0	8	0.1%
71	1	0	0	0	1	0.0%
Total	1367	107	125	10	1609	11.2%

Note:

^1^3 Records with suspicious or malignant carcinoma are not involved in the table.

^2^In our study HPV type 2, 3, 7, 10, 27, 28, 29, 34, 57, 78, and 94 could not be detected so there were no results for Species 2, 4 and 12. Moreover, HPV type 7 from species A8 and HPV 34 from species A11 were removed because the Luminex assay could not detect them.

^3^Records with pap test results unsatisfactory are not included in the table.

#### Age specific

**Figure 2:**
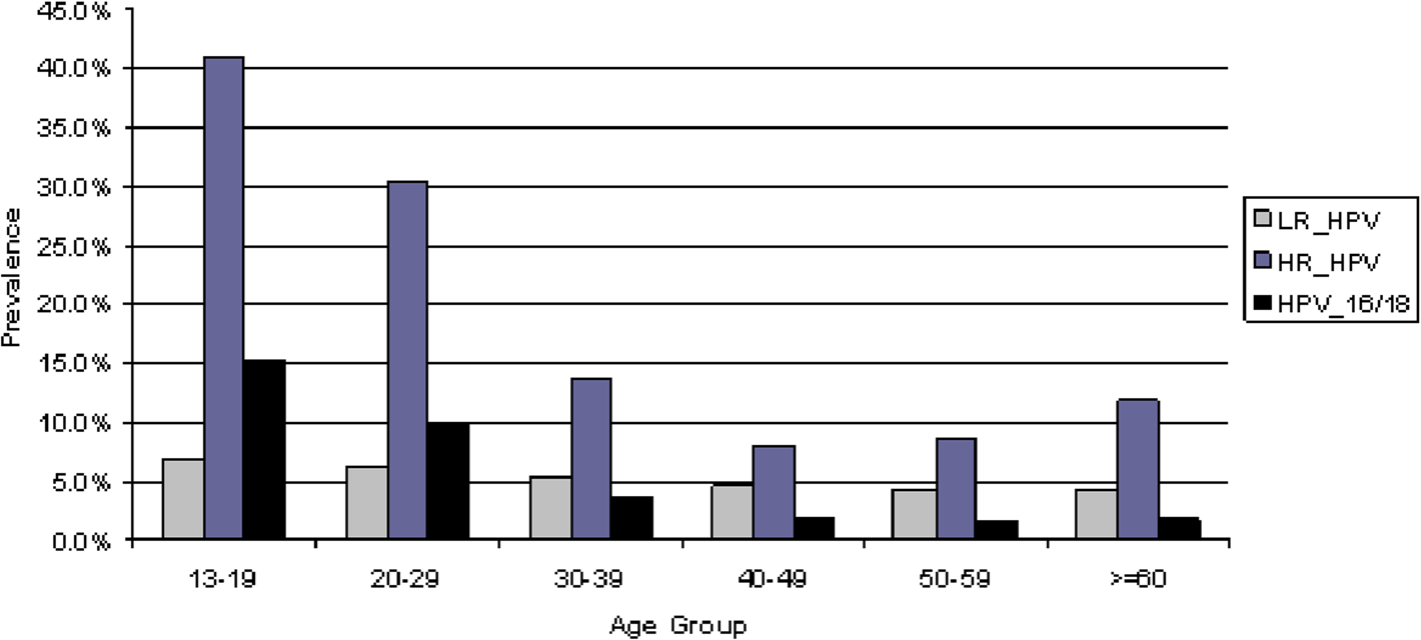
Proportion of HPV types by agegroup, in NWT, Nunavut, Labrador and Yukon, Canada.

**Figure 3:**
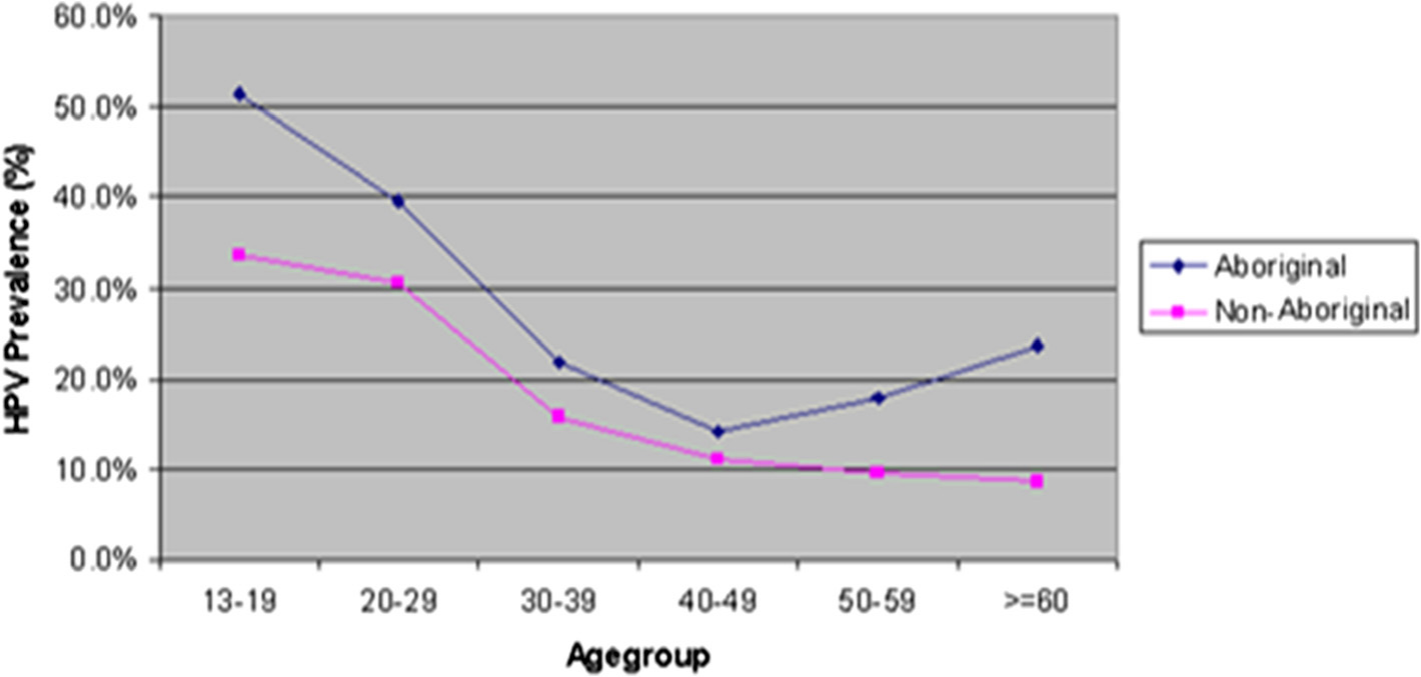
**HPV prevelance by agegroup among Aboriginal and Non-Aboriginal women in NWT**, **Nunavut Labrador and Yukon**, **Canada**.

The age specific HPV prevalence rate ranged between 13% and 48% (Figure 2). The highest prevalence of HR HPV infection and HPV 16/18 was among women aged less than 20 years, with decreasing prevalence until age group 40–49. HPV prevalence was higher in the Aboriginal than the non-Aboriginal women in each age group; in particular among women aged 50 or over (Figure 3). It is of note on the trend that the prevalence of HPV increased again after age 50 thus forming a U-shaped curve in the Aboriginal women, but following a monotonic decrease with age in the non-Aboriginal women.

#### Regional and ethnic differences in HPV prevalence

In Table 2, HPV prevalence rate in NWT, Nunavut, Labrador, and Yukon was 23.2%, 31.5%, 21.4%, and 24.5%; respectively. After adjusting by the NWT 2008 census data for women as a standard population AAPRs changed to 22.7%, 28.0%, 21.1%, and 23.5%; respectively. Any type HPV and HR type HPV AAPRs were significantly higher among the Aboriginal than the non-Aboriginal women. However the HPV 16/18 AAPRs were not statistically different by ethnic group across region. In addition the HR HPV prevalence rates accounted for a higher percentage in any HPV infection among the Aboriginal than among the non-Aboriginal population. HPV 16/18 comprised about 30% of all HR-HPV infection in all four regions.

**Table 2:** **HPV infection prevalence rate among women in Northern Canada**, **2008**-**2010**

**Region**	**#**	**Any type**			**HR**_**HPV**			**HPV 16/18**		
		**#**	**%**	AAPR^*^	**#**	**%**	**AAPR**	**#**	**%**	**AAPR**
				**(95% CI)**			**(95% CI)**			**(95% CI)**
NWT	6724	1561	23.2	22.7 (21.5, 24.0)	1205	17.9	17.5 (16.5, 18.6)	352	5.2	4.9 (4.4, 5.5)
Nunavut	4654	1468	31.5	28.0 (26.3, 29.6)	1205	25.9	22.2 (20.8, 23.7)	387	8.3	4.5 (2.8, 6.3)
Labrador^**^	1370	293	21.4	21.1 (18.6, 23.6)	218	15.9	15.8 (13.6, 18.0)	73	5.3	5.5 (4.2, 6.8)
Yukon	1542	378	24.5	23.5 (20.9, 26.1)	284	18.4	17.7 (15.4, 19.9)	96	6.2	6.0 (4.7, 7.3)
Aboriginal	8446	2623	31.1	27.6 (25.5, 29.8)	2104	24.9	21.6 (19.7, 23.5)	630	7.5	6.1 (5.1, 7.0)
Non-Aboriginal	5844	1077	18.4	18.5 (16.2, 20.8)	808	13.8	13.8 (11.8, 15.7)	278	4.8	4.9 (3.9, 6.0)
P^***^				<0.01			<0.01			>0.05

^*^AAPRs (Age-Adjusted Prevalence Rates) were calculated using NWT 2008 standard population.

^**^Labrador data only had area code (residence in Aboriginal area or not) instead of Aboriginal Status information.

^***^P-value is tested for significant difference between Aboriginal and Non-Aboriginal women in each site using Chi-square test.

#### Prevalence by Pap test results

Overall, 4.0% of Pap test results were abnormal (HSIL and LSIL); 4.7% in Aboriginal and 3.1% in non-Aboriginal group (Table 3). Among the women with normal cytology (92.3%), the HPV prevalence rate in the Aboriginal was 26% in comparison to 15% of the non-Aboriginal group. However, among women with abnormal Pap test, the HPV prevalence was 91.6% in the Aboriginal versus 82.9% in the non-Aboriginal group. Stratified analyses on HPV infection by cytological results in women aged <30 and aged 30 or over among the Aboriginal and the non-Aboriginal showed that distributions of HPV prevalence by cytological result were similar in different ethnic and age groups. In particular, the relative prevalence of HPV 16/18 among HSIL was not significantly different in Aboriginal (46.9) vs Non-Aboriginal women (50.0), as shown in Table 3. Among women with normal cytology 419 (5.4%) and 182 (3.4%) were infected with HPV 16/18 in the Aboriginal and non-Aboriginal group, respectively. The corresponding numbers for HR HPV other than 16/18 infection were 1107 (14.4%) and 401 (7.4%), while the numbers for all HPV infection were 2004 (26%) and 829 (15%), respectively.

**Table 3:** Cytological results by HPV types in Northern Canada

Cytology^*^	**Overall**		**Any type**		**HR**_**HPV**		**LR**_**HPV**		**HPV**_**m**		**HR**_**m**		**HPV 16/18**	
	**N**	%^**^	**N**	%^***^	**N**	%^***^	**N**	%^***^	**N**	%^***^	**N**	%^***^	**N**	%^***^
Aboriginal	8428	100	2617	31.1	2098	24.9	219	2.6	885	10.5	418	5.0	626	7.4
Normal	7693	91.3	2004	26.0	1526	19.8	478	6.2	617	8.0	261	3.4	419	5.4
ASC-US	294	3.5	241	82.0	219	74.5	22	7.5	92	31.3	48	16.3	77	26.2
LSIL	279	3.3	256	91.8	239	85.7	17	6.1	142	50.9	83	29.7	73	26.2
HSIL	113	1.3	103	91.2	103	91.2	0	0.0	28	24.8	24	21.2	53	46.9
unsatisfactory	49	0.6	13	26.5	11	22.4	2	4.1	6	12.2	2	4.1	4	8.2
														
Non-Aboriginal	5765	100	1060	18.4	795	13.8	265	4.6	313	5.4	148	2.6	272	4.7
Normal	5404	93.7	829	15.3	583	10.8	246	4.6	219	4.1	89	1.6	182	3.4
ASC-US	110	1.9	72	65.5	64	58.2	8	7.3	30	27.3	19	17.3	26	23.6
LSIL	133	2.3	110	82.7	103	77.4	7	5.3	46	34.6	27	20.3	38	28.6
HSIL	48	0.8	40	83.3	39	81.3	1	2.1	16	33.3	13	27.1	24	50.0
unsatisfactory	70	1.2	9	12.9	6	8.6	3	4.3	2	2.9	0	0.0	2	2.9

Note:

^*^3 records with suspicious or malignant carcinoma are not involved in the table.

^**^Overall shown column percentage.

^***^Type specifics shown row Percentage.

#### Impact of HPV on cervical abnormalities

After adjusting for Aboriginal status, age, and region, the logistic regression models showed that the ORs for cervical abnormalities associated with any type HPV, LR_s, HR_s, only multiple LR infections, only multiple HR infections and mixed HR and LR infections were 28.9, 5.3, 29.4, 6.2, 71.9, and 35.4, respectively, and that HPV infection accounted for 85.7% of the cervical abnormalities with 80.6% attributable to high risk HPV infection and 32.5% attributable to HPV 16/18 infection (Table 4).

**Table 4:** **Logistic regression analysis of the association between HPV infection and cervical abnormality in NWT**, **Nunavut**, **Labrador and Yukon**, **Canada**

	**HPV Infection**	**Pap test results**		**Adjusted OR (95%CI)**	**ARF**	**PAR**
		**Normal**	**LSIL/HSIL**			
Ref.	HPV Negative	10488	65			
	Any Type HPV	2891	517	28.89 (22.24, 37.53)	96.5	85.7
	Single-HR	1413	260	29.45 (22.30, 38.90)	96.6	43.2
	Single-LR	630	21	5.30 (3.22, 8.74)	81.1	2.9
	Multiple-HR	354	151	71.93 (52.55, 98.47)	98.6	25.6
	Multiple-LR	114	4	6.16 (2.19, 17.31)	83.8	0.6
	HR/LR mix	380	81	35.35 (25.01, 49.98)	97.2	13.5
	Total	13379	582			
						
	HPV16 or 18	614	193	50.82 (37.85, 68.23)	98.0	32.5
						
Ref.	Single-LR	630	21			
	HR/LR mix	380	81	6.47 (3.93, 10.66)	84.5	11.8
				PAR for HR HPV infection: 80.6		

Multiple-HR is HR multiple only.

Adjusted model is adjusted using Age-group, Region, and Aboriginal status. (Labrador is using Aboriginal Area as Aboriginal variables).

## Discussions

This study recruited most of the women aged 18–69 (and most of the incidental consultations for the 14–17 age group) who attended routine or scheduled pap screening over an approximately one year time period.

The prevalence of overall HPV infection was 25.2% which was higher than in most countries [24-27] but similar to Denmark [28] and the US [29]. HPV prevalence rates in both the Aboriginal (31.6%) and non-Aboriginal women (18.9%) in this study were slightly higher than those in other studies in Aboriginal [2,15] and non-Aboriginal [30,31] populations in Canada. The proportion of HR types among the HPV-positive women was lower than in British Columbia and Ontario studies but higher than in a Quebec study [2,30,31].

Similar to most European and Canadian studies the two most common HPV types detected in Northern Canada were HPV-16 and 31 [28,32-36]. The HPV 16/18 prevalence in the study was relatively lower than all similar studies in Canada [2,30,31,35], but comparable to studies in Europe and the US [25,26,28,29,37]. In this study HPV 16/18 comprised about 31% of all HR-HPV infections. Several HR HPV types other than HPV 16/18, such as HPV 31, 39, 45, 51, 59 and 66 were relatively common in Northern Canada.

The prevalence analysis shows that HPV prevalence was highest among women aged less than 20 years, which is consistent with other studies [27,30,31,38,39]. This finding confirms what is already known on the natural history of HPV infection, which is characterized by higher infection rates after sexual initiation. In agreement with published data [2,40], the age adjusted HPV prevalence rate in the Aboriginal group was higher than that in the non-Aboriginal group. Inconsistent trends in HPV prevalence by age were noted in older women, with a decrease or plateau of HPV prevalence among the non-Aboriginal group, whereas the Aboriginal group showed an increase of HPV prevalence (U-shaped age-specific HPV prevalence distribution). This has been observed in the other studies in Canada [2,15,30,31] and from Costa Rica and other Latin American countries [41,42], with the lowest prevalence among the age group 40–49. However, from most of the European literature, with the exception of the former Soviet Union [33], a decrease in HPV prevalence after age 20 and a levelling off after age 45, has been reported [25,26,28,42]. Further study needs to appreciate what role that social, cultural and lifestyle factors, as well as environmental determinants such as access to care may play in explaining this difference in age distribution.

Noteworthy is that, despite the difference in prevalence of HPV types and cytological abnormalities between Aboriginal and Non-Aboriginal women, the proportion of HPV 16/18 in high grade lesion is about 50% in both groups. This is very similar to the average proportion of HPV 16/18 in HSIL in North America and worldwide [13]. Since HSILs are the precursors of invasive cancer, these results suggest that the current HPV vaccines should be equally applicable to Aboriginal and Non-Aboriginal women.

Currently routine Pap testing is one of the pillars to prevent cervical cancer. Other studies have found similar Pap test abnormality rates [2,30]. HPV prevalence was higher in those women aged < 30 than in older women across the spectrum of abnormal Pap results. This is consistent with reports showing that HPV infection is common in young women and most often transient [43-46]. It is expected that women aged 30 years and older experience more persistent HR infections than younger women and that HPV-DNA testing would be able to triage this group for further follow-up.

Among participants with a normal pap test result, 26% of any type HPV infection among the Aboriginal group and 15% among the non-Aboriginal women (Table 3) translated into a 28 times higher risk of cervical abnormality than those with HPV negative (Table 4). By the same token, among 5.4% of the Aboriginal women and 3.4% of the non-Aboriginal women with both normal Pap test results and HPV 16/18 infection, they have a 50 times higher risk of cervical abnormality than those who are HPV negative. Interestingly, similar to our previous NWT report [47], results seem to indicate an additive risk model for multiple HPV infections. This information may be useful in considering HPV testing in cervical cancer screening programs in Northern Canada.

There are potential limitations in the study. Firstly, no PCR protocol is ideal for all types of HPV detection. Any of the above comparisons in prevalence rates must be taken with caution as we have used the most recent IARC classification for HR HPV and a novel (Luminex) detection method as sensitive as Linear Array but able to detect more types than other previously reported technologies such as GP5+/6+, Elisa, or Hybrid Capture [17,18]. Secondly, this study represents HPV infection at various time points in the different regions, and does not represent the proportion of women at risk for disease or cancer related to HPV infection. Of note, there was no full regional coverage of the targeted populations during the study period. Different regions had different recruitment methods and therefore rates. Due to an “opt-in” process, the coverage for the Yukon sites lower than the other regions, and one thus should be cautious in interpreting the prevalence rates of the study population for Yukon. In addition, the Aboriginal status was not available in Labrador. To estimate HPV prevalence by ethnic group in the entire North in Canada, we used residence for Aboriginal or non-Aboriginal area instead as the purpose of the study was to understand the severity of HPV prevalence in Northern Canada and each northern region. Although we provided both crude rates and age adjusted rates for HPV infection, it still needs to be cautious to make comparison by region due to substantially different data collection processes. Finally, it’s worth noting that routine screening is not recommended by standard guidelines for young women aged 14–18. In addition Pap screening is not mandatory for anyone; e.g., NWT has their own guidelines [48] and Yukon follows a guideline from British Columbia. Therefore, there may be a selection bias toward the young age group in data analysis. Nevertheless, the overall analysis was conducted with and without the 14–18 years age group and the results remained similar. We suspect also that there may be a potential bias affecting participation by Aboriginal versus non-Aboriginal people.

## Conclusions

In Northern Canada, the prevalence of HPV infection was higher than other Canadian areas and varied by region. Between the Aboriginal and the non-Aboriginal group, the age specific distribution rates of HPV infections appear to be different; however, the effect of HPV infection to cervical dysplasia was similar. The relatively high prevalence of HR-HPV other than HPV 16/18, in particular among the Aboriginal group, call for further study to understand the impact of specific predictors [49]. This study provides baseline information on HPV prevalence that may assist in surveillance and evaluation systems to track and assess HPV vaccine programs.

## Abbreviations

AAPR: Age-adjusted prevalence rate; ASC-H: Atypical squamous cells where HSIL could not be excluded; ASC-US: Atypical squamous cells of undetermined significance; ARF: Attributable risk fraction; CI: Confidence interval; HPV: Human papillomavirus; HPV_m: Two or more types of HPV infection; HPV_s: a Single type of HPV infection; HR: High risk; HR_m: Two or more types of high risk infection; HR_s: a Single type of high risk infection; HSIL: High-grade squamous intraepithelial lesion; LR: Low risk; LSIL: Low-grade squamous intraepithelial lesion; NML: National microbiology laboratory; NWT: Northwest territories; OR: Odds ratio; Pap: Papanicolaou test; PAR: Population attributable risk fraction; PCR: Polymerase chain reaction.

## Competing interests

There is no conflict of interest related to this work from any author.

## Authors’ contributions

YJ: linked the datasets, analyzed the data, drafted the manuscript. PB: participated in the data collection, data analysis and results interpretation. AS: carried out all HPV DNA typing tests, revised the manuscript and helped to finalize the manuscript. YM: designed the study and made the substantial contributions to interpretation of data. YAL: involved in the majority of the project management work for NWT and Yukon. JAL: involved in data interpretation and helped revise the manuscript. SC: carried out the data collection in NWT and involved in the results discussion. AC: carried out the study in NWT site and involved in the results discussion. KK: carried out the study in NWT site and involved in the data collection and linkage. BH: carried out the data collection in Yukon site and provided with the critical review of the results. IS: initiated and managed the data collection in Nunavut. MA-R: initiated and carried out the data collection in Labrador-Grenfell Health, and involved in the results discussion. GJ: carried out Pap test for samples from NWT and Nunavut. JL: carried out Pap test for samples from Yukon. SR: carried out Pap test for samples from Labrador and involved in the results discussion. TW: involved in the data collection in NWT, Nunavut, and Labrador. AD: participated in the analysis of data and the critical review of the results. GJ: participated in conducting HPV type-specific surveillance and linkages to cytology results in Nunavut, involved in data interpretation and helped revise the manuscript. ST: involved in data interpretation and helped revise the manuscript. HM: performed the study and revised the manuscript. All authors read and approved the final manuscript.
